# The burden of depressive disorder among the global 10–24 age group and the construction of an early risk factors model

**DOI:** 10.3389/fpsyt.2025.1594074

**Published:** 2025-06-16

**Authors:** Yangyi Guo, Hongxin Lu, Aidi Chen, Jing Guo, Yuyang Lai, Zhengyou Lu

**Affiliations:** Department of Clinical Laboratory, The Third Hospital Of LongYan, LongYan, Fujian, China

**Keywords:** depressive disorder, machine learning, S100β, NSE, GBD

## Abstract

**Objective:**

To understand the global trends in depression and identify potential early risk factors for its detection.

**Methods:**

This study is the first to integrate the 2021 Global Burden of Disease (GBD) data with machine learning techniques to explore the risk factors of adolescent depression. A machine learning-based model was constructed, and SHAP (SHapley Additive exPlanations) plots were utilized for interpretive analysis.

**Results:**

From 1990 to 2021, the incidence and disability-adjusted life years (DALYs) of depression continued to rise globally among the 10–24 age group, particularly in high socio-demographic index(SDI) regions. Greenland, the United States of America, and Palestine had the highest rates of depression globally. Among the eight machine learning models evaluated, random forest (RF) proved to be the most reliable. SHAP analysis revealed that elevated levels of S100β (0.330), NSE (0.060), and PLT (0.031) significantly increased the risk of depression.

**Conclusion:**

Our study shows an increasing trend of depression in the global 10–24 age group. Additionally, elevated S100β, NSE, and PLT are identified as key risk factors for depression.

## 1 Introduction

Depression disorder is not merely a short-term mood disturbance; it is a chronic and potentially recurrent mood disorder. Common symptoms include persistent low mood, loss of interest, difficulty concentrating, changes in appetite, fatigue, sleep disturbances, and self-criticism. With the rapid global economic development, depression among adolescents has become an increasingly serious issue, drawing public attention. According to data from the World Health Organization (WHO), suicide is the fourth leading cause of death worldwide, with depression being the primary contributor (1). Some studies predict that by 2030, depression may become the leading cause of disease burden among adolescents (2). Therefore, there is an urgent need to adopt effective strategies to address the growing burden of depression, particularly through early detection and intervention to reduce its long-term impact on the adolescent population.

Currently, traditional diagnostic methods for depression rely on the patient’s clinical symptoms, symptom history, and diagnostic criteria. The most commonly used diagnostic tools include the Diagnostic and Statistical Manual of Mental Disorders, Fifth Edition (DSM-5) (3) and the International Classification of Diseases, 11th Edition (ICD-11) by the World Health Organization. However, the accuracy of these assessment tools is influenced by participants’ compliance and the subjective interpretation of healthcare professionals. Additionally, the consistency of diagnosis is challenging to guarantee due to differences in economic, cultural, and healthcare levels. Furthermore, these diagnostic methods exhibit several limitations in the context of adolescent mental health diagnosis: 1. The behavioral patterns and severity of impairment in adolescents often differ from those in adults. 2. The social and environmental context of adolescent development is not adequately considered. 3. The diagnostic process places excessive emphasis on internal factors, neglecting the unique characteristics of the adolescent population. 4. The distinction between normal and abnormal behavior is sometimes subjective and arbitrary. Therefore, this study aims to address these gaps and propose a new diagnostic framework or approach, with the goal of improving diagnostic accuracy for the adolescent population.

In recent years, machine learning (ML) technology has been widely applied in the detection of depression (4, 5). Compared to traditional methods, machine learning enables more precise analysis of complex relationships between variables. Through big data analysis, it effectively predicts the risk of depression (6). When handling complex data, machine learning can filter out more valuable information and provide more reliable judgments.

This study aims to explore the burden of depression in the 10–24 age group and, through objective blood biomarkers, exclude some subjective factors. By combining machine learning analysis, we seek to identify potential risk factors for depression. Through this approach, we hope to provide more effective support for the early detection and intervention of depression.

## 2 Methods

### 2.1 Data source

The data for this study were obtained from the Global Burden of Disease (GBD) 2021 study, coordinated by the Institute for Health Metrics and Evaluation (IHME). The GBD study collects data from a wide range of sources, including censuses, vital registration systems, household surveys, health service use statistics, disease registries, and published scientific literature. All data inputs were standardized and adjusted to improve comparability across time and locations. Data were processed using the GBD’s standardized analytical tools, including cause of death ensemble modeling (CODEm), DisMod-MR 2.1 for non-fatal estimation, and spatiotemporal Gaussian process regression (ST-GPR). Detailed data processing protocols are publicly available at the IHME GBD methodology website (http://www.healthdata.org/gbd/). IHME follows the University of Washington’s website terms and conditions: http://www.washington.edu/online/terms/.

In addition, for this study, the diagnostic criteria for 250 adolescent depression patients and 200 healthy controls from Longyan Third Hospital in China followed the depression diagnostic standards from DSM-5, consistent with those in the GBD database. All diagnoses of depression were confirmed by experienced clinical psychologists through interviews and clinical assessments, meeting the following criteria:

Unipolar Depression: Patients must have experienced significant symptoms of mood depression or loss of interest in the past two weeks, with other common symptoms including: 1. Reduced concentration and attention; 2. Lowered self-esteem; 3. Guilt feelings and sense of worthlessness (even in mild episodes); 4. Pessimism about the future; 5. Thoughts or behaviors of self-harm or suicide; 6. Sleep disturbances; 7. Reduced appetite.

Bipolar Disorder: Patients in the current depressive episode must also meet the diagnostic criteria for bipolar disorder, including at least one history of manic or hypomanic episodes. Bipolar I Disorder: At least one manic episode (may be accompanied by a depressive episode), with no threshold for hypomanic episodes (mania alone is sufficient for diagnosis). Bipolar II Disorder: At least one hypomanic episode + at least one depressive episode, with no history of manic episodes.

Healthy Control Group: No symptoms or history of depression, excluded based on the Hamilton Depression Scale (HAMD) and Hamilton Anxiety Scale (HAMA).

In addition, this study did not compare the small sample data with the GBD dataset. We used the GBD dataset to study the epidemiological trends of depression in the adolescent population and understand the increasing burden of depression in this group. Therefore, the development of machine learning methods provides more effective support for the early detection and intervention of depression in adolescents.

According to the 10 EPV principle (which requires at least 10 samples per predictor variable), with 450 samples, 250 events, and 15 predictor variables, the EPV = 16.67, which is well above the commonly recommended 10 EPV standard (7).

### 2.2 Data collection

Depression disorder is not just a mental disorder; its occurrence is closely related to various physiological and biochemical factors. It may be associated with neural damage and neuroinflammation (8), thyroid function (9), the body’s metabolism (10), inflammatory responses (11), and cardiovascular factors (12). Therefore, data were collected from laboratory test results, including age, gender, NSE, S100β, FT3, FT4, TSH, BUN, CRE, LDH, CK, CK-Mb, WBC, RBC, HGB, PLT, among other parameters. The detection of NSE, S100β, FT3, FT4, and TSH is performed using chemiluminescence immunoassay (CLIA), utilizing the Snibe MAGLUMI X8 analyzer (https://www.snibe.com/en/product/CLIA_analyzer/40.html) and its associated reagents. The measurements of BUN, CRE, LDH, CK, and CK-Mb are conducted using colorimetric methods, with the Mindray BS-2000M analyzer(https://www.mindray.com/en/products/laboratory-diagnostics/chemistry/large-test-volume/bs-2000m).The analysis of WBC, RBC, HGB, and PLT is carried out using impedance and flow cytometry methods, employing the Sysmex XS-1000i analyzer (https://www.sysmex-wca.com/products/products-detail/xs-1000i/) and its corresponding consumables. All participants had no organic damage or underlying diseases. The diagnosis of depression was based on DSM-5, and the severity of depressive symptoms was assessed using the 17-item Hamilton Depression Rating Scale (HAMD-17).Level 1: Mild depressive episode; Level 2: Moderate depressive episode; Level 3: Severe depressive episode without psychotic features; Level 4: Severe depressive episode with psychotic features (13). And it also applies to this region (14, 15). This study has been approved by the Ethics Committee of The Third Hospital Of LongYan, and informed consent has been obtained from the patients or their guardians.

### 2.3 Testing methods

CLIA:CLIA is an immunoassay technique based on the generation of light signals through chemical reactions. In CLIA, the binding of antibodies to antigens is marked with chemiluminescent substances (such as enzyme labels or other chemiluminescent probes). Under certain reaction conditions, the label emits detectable light. During the reaction, when the antigen (target substance) binds to the antibody, light signals are generated through reactions with enzymes or other chemical reagents. These light signals are quantified using a photometer or chemiluminescence analyzer.

Colorimetric methods:Colorimetric methods are a common analytical technique based on the relationship between the color change in a solution and the concentration of the sample. In colorimetric analysis, a reagent is typically used that reacts with the target molecule to produce a color change. When the target molecule (such as proteins, enzymes, or other chemicals) reacts with the reagent, the color of the solution changes. This change can be measured using instruments such as a spectrophotometer to obtain absorbance values. By using a standard curve relating absorbance to known concentrations, the target substance in the sample can be quantified.

Impedance method:The impedance method analyzes changes in impedance when cells or particles are exposed to an electric field. The movement and morphological changes of cells in the electric field affect the conductivity, and changes in impedance can provide information about the size, shape, number, and biological activity of the cells. The impedance method is suitable for cell counting, cell biology research, and cell viability testing. Typically, electrodes or sensors make contact with the cells in the sample to measure changes in resistance or capacitance.

Flow cytometry:Flow cytometry is an efficient single-cell analysis technique that analyzes scattered light and fluorescence signals produced when cells pass through a laser beam. When cells are suspended in liquid and pass through a light source, they interact with the light; the scattered light provides information about the cell’s size and granularity, while fluorescence signals can be used to detect specific molecules or antigens labeled within the cells. Flow cytometry allows high-speed, multi-parameter analysis of cells and can be used for cell counting, cell cycle analysis, immunophenotyping, and cell sorting.

### 2.4 Data processing

In the data analysis, the Kolmogorov-Smirnov test was used to assess the normality of the data. If the p-value is less than 0.05, the data are considered not to follow a normal distribution. normally distributed variables were described using absolute numbers and percentages, means, and standard deviations, while non-normally distributed variables were represented by medians and interquartile ranges. The analysis of categorical variables was performed using the chi-square test or Fisher’s exact test, and the comparison of continuous variables was done using the two-sample t-test or Mann-Whitney U test. All hypothesis tests were two-sided, and the significance level was set at α = 0.05. If the p-value is less than 0.05, the null hypothesis is rejected, and the result is considered statistically significant. A linear regression model was used to calculate the annual average percentage change (EAPC) value and its 95% confidence interval (CI). The criteria for determining trends were as follows: if EAPC>0, it indicated an increase in the variable over time; if EAPC<0, it indicated a decrease over time (16).

### 2.5 Model construction and evaluation

The 450 patients were randomly divided into a training set and a validation set in a 7:3 ratio. The ratio of 7:3 is commonly used as a rule of thumb when splitting a dataset into training and test sets (17). A univariate logistic regression was used, and the forest plot was constructed based on the training set. Eight machine learning algorithms were used to construct predictive models, including Decision Tree (DT), K-Nearest Neighbors (K-NN), Light Gradient Boosting Machine (LightGBM), Logistic Regression (LR), Naive Bayes (NB), Random Forest (RF), Support Vector Machine (SVM), and Extreme Gradient Boosting (XGBoost). The performance of each model was evaluated by comparing metrics such as AUC, F1 score, sensitivity, specificity, recall, and accuracy.Finally, this study used Random Forest for model construction. The parameters for the random forest are as follows: nestimators=500, nodesize=1, maxfeatures=3. For the development of the machine learning model, 5-fold cross-validation was used to train the model, which helped identify the optimal hyperparameters.

To further understand the prediction principles of the models, Shapley Additive Explanations (SHAP) plots were used. SHAP values provide a universal metric for explaining each feature’s contribution to the model’s predictions. By analyzing the SHAP value density scatter plots for each feature, the influence of each variable on the model output from the validation set was determined. The ranking of variables was based on the total SHAP values across all samples. All data analyses were performed using the statistical software R (version 4.4.2).

## 3 Results

### 3.1 Overall trend

From 1990 to 2021, the incidence of depression among the global population aged 10–24 years increased from 3085.52 (2307.81–4105.56) to 3909.88 (2841.30–5171.10), while the DALYs increased from 454.05 (291.66–657.43) to 567.77 (359.80–828.13). The Annual Average Percentage Change (EAPC) for incidence and DALYs were 0.16 (-0.06–0.39) and 0.21 (0.02–0.41), respectively.

**Figure 1:**
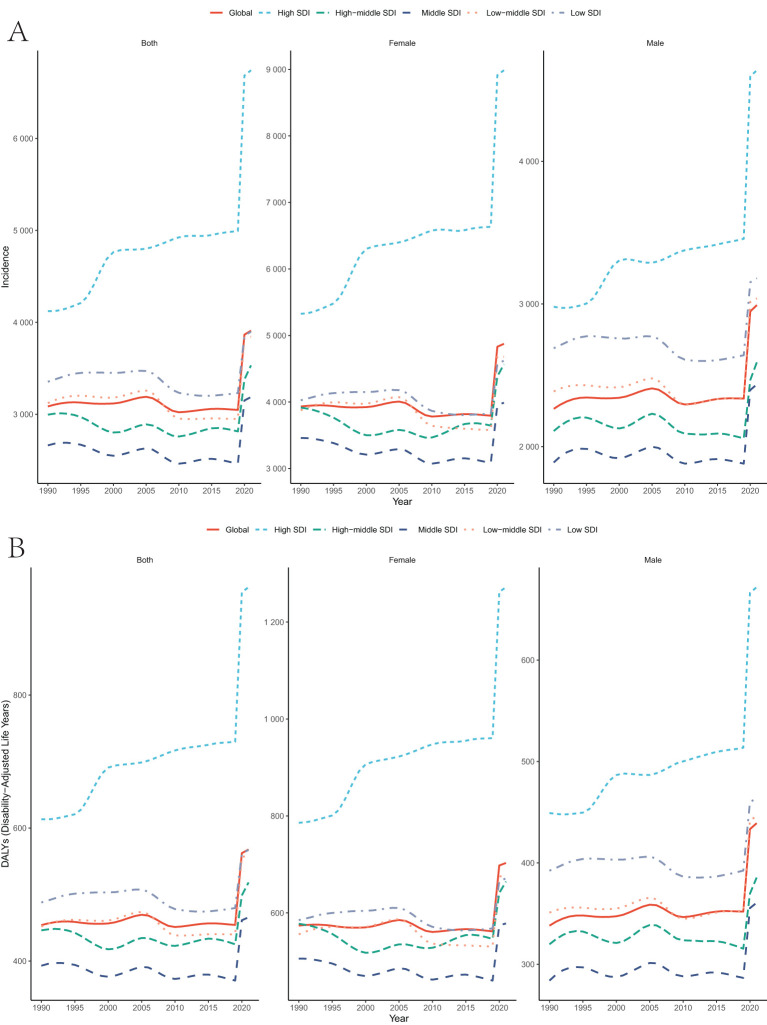
Temporal trend of incidence,DALYs rates for the burden of depressive disorders(10–24 year) by sex, SDI from 1990 to 2021. (Based on the GBD dataset).

In terms of gender distribution, the number of female patients was higher than that of males, but the increase in male incidence was more pronounced. The EAPC for males was 0.27 (0.06–0.49) for incidence and 0.31 (0.12–0.50) for DALYs, which was significantly higher than the corresponding values for females, which were 0.11 (-0.12–0.33) and 0.16 (-0.03–0.36), respectively. Regarding the Socio-Demographic Index (SDI), the incidence and DALYs in high-SDI regions were significantly higher than in other regions, with the EAPC growth rate far exceeding that of low and middle-SDI regions, which were 0.99 (0.74–1.24) and 0.93 (0.71–1.15), respectively (**Table 1**). Specifically, high-SDI regions have shown consistent growth since 1990, with an increase far exceeding other SDI-level regions, which is in line with the EAPC trend. From a time trend perspective, the global incidence and DALYs of depression remained stable and fluctuating until 2019. However, since the outbreak of the COVID-19 pandemic in 2019, this trend has significantly increased (**Table 1**, **Figure 1**).

**Table 1:** Incidence Rates, DALYs Rates of depressive disorders(10–24 year) by sex, SDI in 1990 -2021 (Based on the GBD dataset).

Categories	Incidence			DALYs		
	Rates in 1990 (95% UI)	Rates in 2021 (95% UI)	1990-2021 EAPC (95% CI)	Rates in 1990 (95% UI)	Rates in 2021 (95% UI)	1990-2021 EAPC (95% CI)
Global	3085.52 (2307.81-4105.56)	3909.88 (2841.30-5171.10)	0.16 (-0.06,0.39)	454.05 (291.66,657.43)	567.77 (359.80,828.13)	0.21 (0.02,0.41)
SEX						
Male	2265.25 (1684.16,3013.94)	2991.66 (2155.46,3978.65)	0.27 (0.06,0.49)	338.12 (216.20,494.06)	439.14 (279.52,646.92)	0.31 (0.12,0.50)
Female	3932.73 (2961.20,5221.66)	4875.11 (3571.80,6378.79)	0.11 (-0.12,0.33)	573.79 (369.60,827.25)	702.98 (446.98,1020.95)	0.16 (-0.03,0.36)
SDI						
High SDI	4122.80 (3206.03-5253.29)	6741.92 (5069.35-8619.87)	0.99 (0.74,1.24)	613.19 (399.96,879.93)	962.21 (627.83,1410.62)	0.93 (0.71,1.15)
High-middle SDI	2995.89 (2305.46-3891.48)	3531.73 (2610.13-4628.51)	0.02 (-0.19,0.23)	446.24 (290.60,643.84)	518.19 (325.38,769.50)	0.08 (-0.09,0.26)
Middle SDI	2661.98 (1988.23-3560.56)	3187.19 (2362.04-4232.92)	-0.01 (-0.25,0.22)	393.03 (252.02,571.57)	465.82 (297.34,684.50)	0.05 (-0.15,0.26)
Low-middle SDI	3121.68 (2303.42-4247.04)	3844.53 (2755.73-5175.89)	-0.05 (-0.31,0.21)	452.01 (287.02,665.89)	558.01 (352.07,812.73)	0.06 (-0.16,0.28)
Low SDI	3354.24 (2359.71-4551.50)	3897.59 (2708.94-5303.65)	-0.08 (-0.27,0.11)	488.12 (303.33,717.58)	567.10 (343.50,835.76)	-0.01 (-0.18,0.15)

EAPC, estimated annual percentage change; SDI, Sociodemographic Index; UI, uncertainty interval.

a EAPC is expressed as 95% CIs.

**Figure 2:**
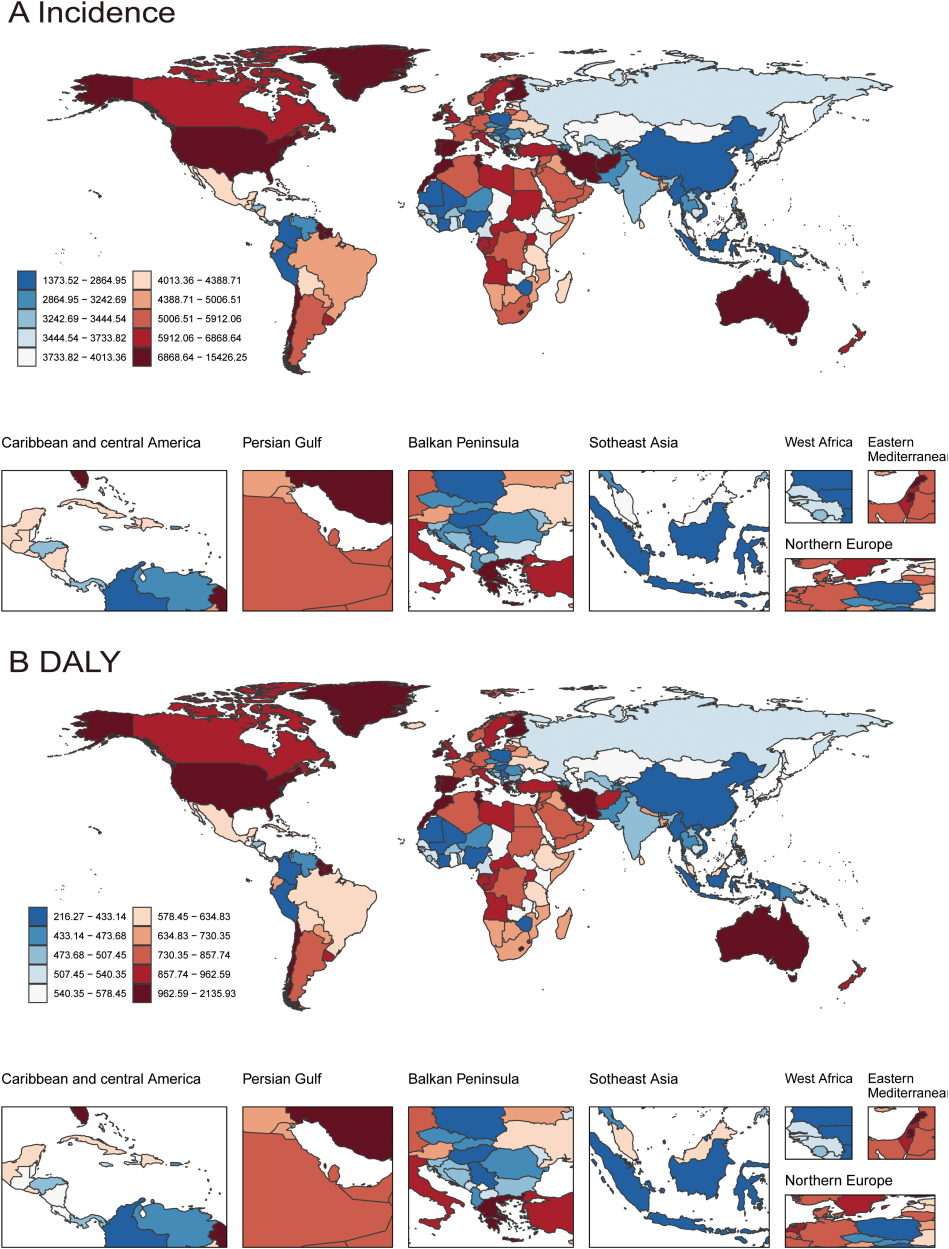
The incidence and disability-adjusted life years (DALYs) rates for depressive disorders(10-24 year) in 204 countries from 1990-2021. (Based on the GBD dataset) **(A)** Incidence; **(B)** DALYs.

At the national level, the top three countries with the highest incidence and DALYs were: Greenland, with an incidence of 15273.51 (10282.59–21274.47) and DALYs of 2114.78 (1289.81–3263.91); the United States of America, with an incidence of 10214.88 (8022.64–12897.19) and DALYs of 1431.52 (969.19–2070.3); and Palestine, with an incidence of 9287.97 (6090.49–13597.09) and DALYs of 1311.01 (765.05–2081.03) (**Figure 2**; **Supplementary Table S1**).

### 3.2 General information of patients

A total of 450 patient data were collected in this study, including 200 non-depressed individuals (44.44%) and 250 depressed patients (55.56%). The gender distribution of patients was 206 males (45.78%) and 244 females (54.22%). The results indicated that the levels of NSE, S100β, WBC, and PLT in depressed patients were significantly higher than those in the non-depressed group, while FT3, BUN, and HGB levels were significantly lower in the depressed group (**Table 2**). Additionally, the levels of NSE and S100β increased progressively with the severity of depressive symptoms (**Supplementary Table S2**).

**Table 2:** Characteristics of research participants (Based on 450 patients).

Variable	ALL N=450	healthy population N=200	depressive disorders N=250	*P*
Gender:				0.130
Male	206 (45.78%)	100 (50.00%)	106 (42.40%)	
Female	244 (54.22%)	100 (50.00%)	144 (57.60%)	
Age	18.00 (15.00-21.00)	20.00 (17.00-22.00)	16.00 (15.00-18.00)	
NSE	11.50 (9.70-17.17)	10.10 (9.11-11.53)	16.41 (10.93-19.20)	<0.001
S100β	5.71 (2.86-8.57)	2.86 (1.79-3.81)	7.86 (6.19-11.43)	<0.001
FT3	4.47 (4.21-4.79)	4.51 (4.31-4.82)	4.42 (4.12-4.74)	0.002
FT4	17.79 (2.20)	17.62 (2.34)	17.94 (2.07)	0.130
TSH	1.87 (1.31-2.42)	1.72 (1.25-2.44)	1.90 (1.48-2.40)	0.177
CRE	63.00 (54.00-75.00)	63.00 (55.00-76.00)	62.00 (52.00-73.00)	0.083
LDH	165.00 (148.00-186.00)	167.00 (149.00-183.25)	164.00 (147.00-187.00)	0.824
CK	88.00 (65.25-118.00)	90.50 (72.00-118.25)	85.50 (62.25-118.00)	0.186
CK-Mb	11.00 (9.00-13.00)	11.00 (9.47-13.30)	11.00 (8.00-13.00)	0.086
BUN	4.43 (3.83-5.15)	4.62 (4.02-5.28)	4.27 (3.67-4.84)	<0.001
WBC	6.30 (5.38-7.60)	5.97 (5.21-7.06)	6.60 (5.68-7.84)	<0.001
RBC	4.88 (4.51-5.35)	4.88 (4.59-5.30)	4.86 (4.50-5.40)	0.751
HGB	138.00 (129.00-151.00)	140.00 (133.00-152.00)	137.00 (126.00-150.00)	0.007
PLT	264.00 (226.00-303.00)	256.50 (216.00-286.00)	277.00 (236.25-320.75)	<0.001
level:				
0	200 (44.44%)	200 (100.00%)	0 (0.00%)	
1	66 (14.67%)	0 (0.00%)	66 (26.40%)	
2	79 (17.56%)	0 (0.00%)	79 (31.60%)	
3	17 (3.78%)	0 (0.00%)	17 (6.80%)	
4	88 (19.56%)	0 (0.00%)	88 (35.20%)	

Values are expressed as mean ± SD, medians (interquartile ranges), or percentages.

**Figure 3:**
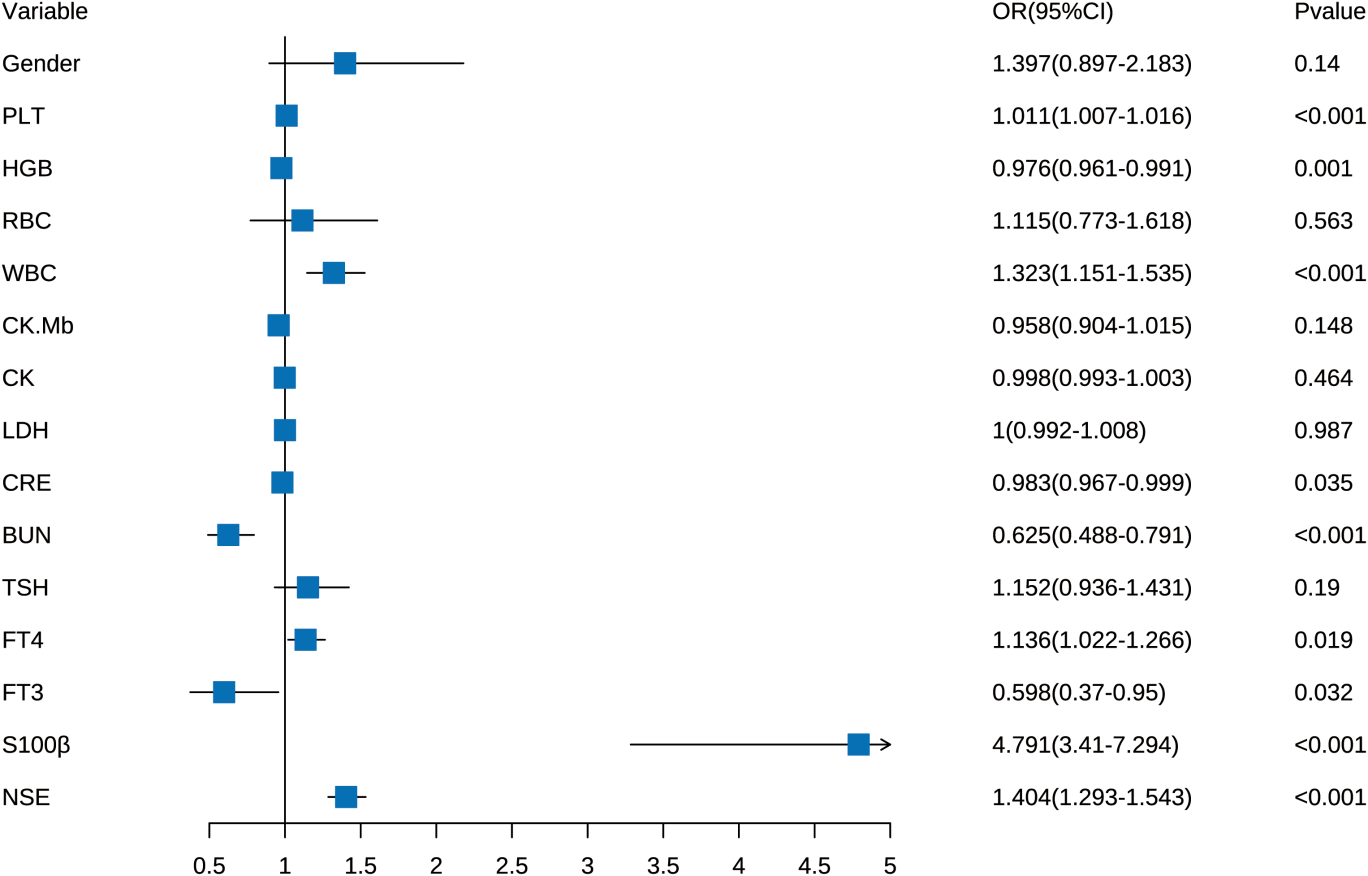
The forest plot of the OR of the selected variables. Forest plot for outcome in Univariate Logistic Regression Analysis. (Based on 450 patients).

Univariate logistic regression analysis showed significant statistical differences between the two groups for PLT (1.011, 1.007–1.016), HGB (0.976, 0.961–0.991), WBC (1.323, 1.151–1.535), CRE (0.983, 0.967–0.999), BUN (0.625, 0.488–0.791), FT3 (1.136, 1.022–1.266), FT4 (0.598, 0.37–0.95), S100β (4.791, 3.41–7.294), and NSE (1.404, 1.293–1.543) (**Figure 3**).

### 3.3 Machine learning model construction and evaluation

**Figure 4:**
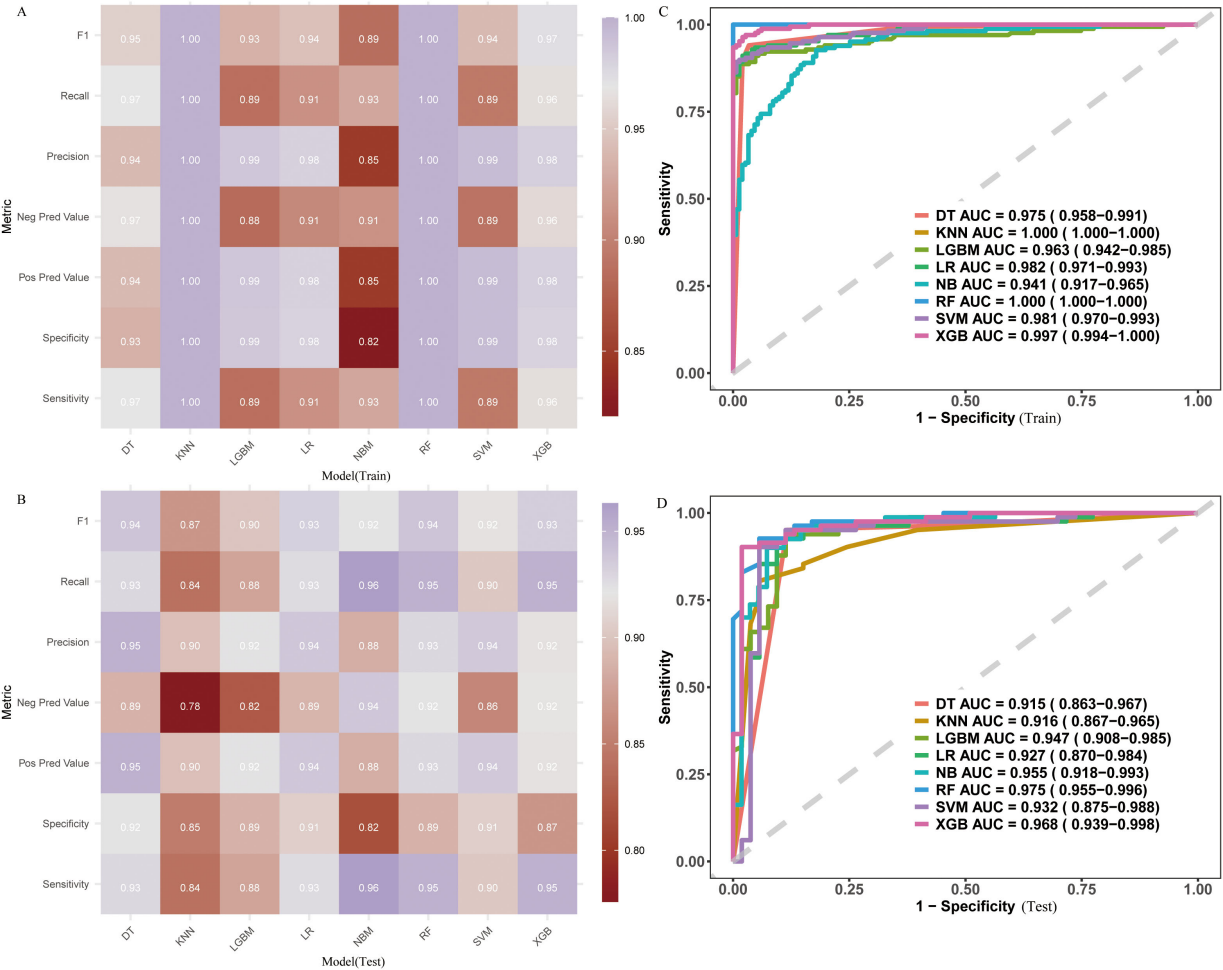
Performance metrics of the predictive model in the training and validation sets (based on 450 patients). **(A)** Training metrics; **(B)** Testing metrics; **(C)** ROC curve for training set; **(D)** ROC curve for testing set.

We applied the nine aforementioned variables to construct eight machine learning models, including DT, K-NN, LightGBM, LR, NB, RF, SVM, and XGBoost. The performance metrics of each model are shown as follows (**Figure 4**). Among all machine learning methods, the Random Forest (RF) model generally performed the best, demonstrating superior performance. Specifically, the RF model achieved the highest levels in F1 score (0.94), recall (0.95), negative predictive value (0.92), and sensitivity (0.95). Other metrics, such as accuracy (0.93), positive predictive value (0.93), and specificity (0.89), also remained at a high level. Ultimately, the RF model’s ROC curve area under the curve (AUC) was the highest, reaching 0.975 (0.955-0.996), indicating that RF might be the most reliable model among the eight machine learning methods, offering a good balance across all evaluation metrics. Additionally, the XGBoost model also exhibited strong performance, with results comparable to RF.

### 3.4 Variable importance and SHAP plot

**Figure 5:**
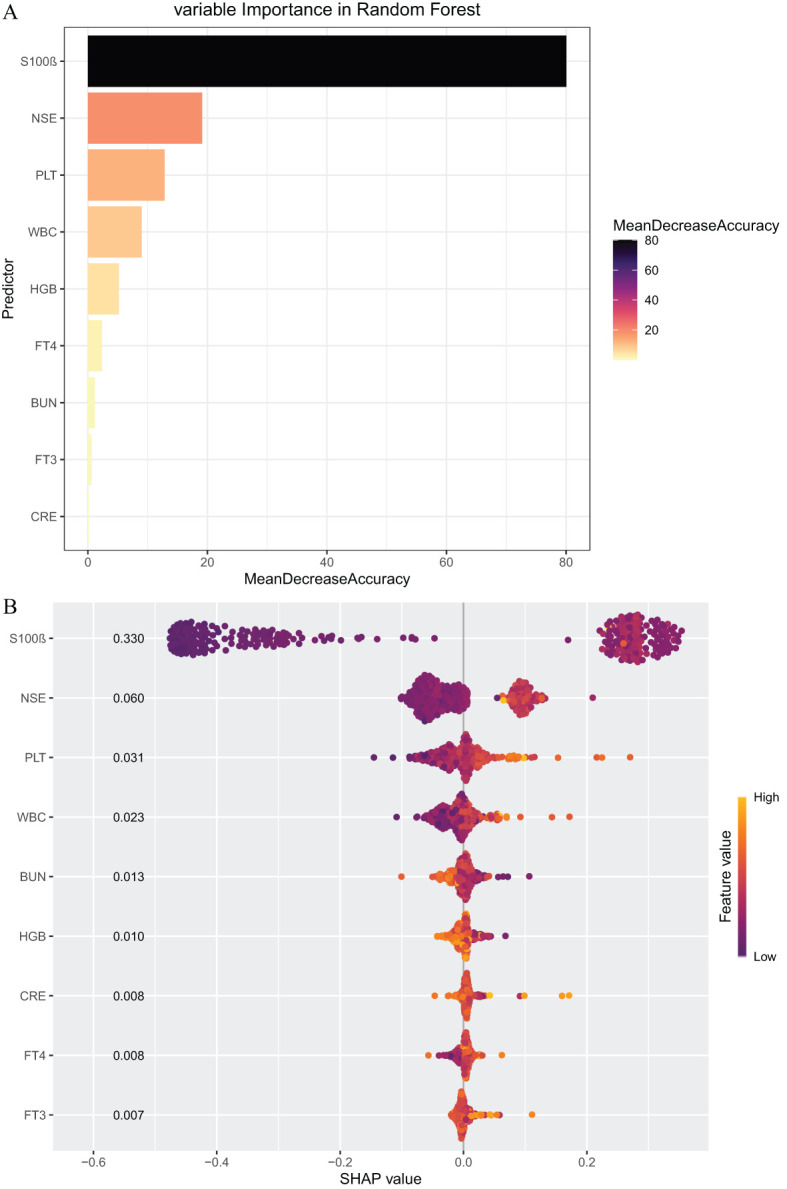
**(A)** Feature Importance of Random Forest (RF). **(B)** A visual interpretation through SHAP plots. In the plot, yellow represents higher feature values, while red indicates lower feature values. (Based on 450 patients).

We ranked the importance of each variable using the RF model and provided a visual interpretation through SHAP plots (**Figure 5**). The results indicated that S100β, NSE, and PLT were the top three most important variables. Specifically, S100β was identified as the most important variable, having the greatest impact on the predictive model, followed by NSE, with both variables showing similar levels of influence.

The SHAP plot provides an in-depth analysis of model predictions. Through the SHAP value density scatter plot for each feature, we can assess the extent of each variable’s influence on the model output for individual samples in the validation set. The variables are ranked according to the total SHAP value sum across all samples. In the plot, yellow represents higher feature values, while red indicates lower feature values. The SHAP values on the x-axis represent the contribution of each variable to the model’s output. Negative SHAP values suggest that the variable has a protective effect against depression, whereas positive SHAP values indicate an increased likelihood of depression. The results showed that the SHAP value for S100β was 0.330, indicating that an increase in S100β significantly raised the risk of depression. The next most influential variables were NSE (0.060) and PLT (0.031).

## 4 Discussion

In this study, we utilized the GBD database to investigate the trends and burden of depression among individuals aged 10–24 from 1990 to 2021. The results indicate a significant upward trend in both the incidence of depression and DALYs in this age group, particularly during the COVID-19 pandemic, when this trend became more pronounced (18, 19). These data reflect the increasing burden of depression among young people aged 10-24. Due to global differences in economy, culture, and education levels, there may be biases in the depression measurement tools, leading to inaccurate assessments (20, 21). Therefore, to detect early signs of depression in the youth population, we adopted blood biomarkers and developed a simple predictive model using eight machine learning methods. The model effectively explained the relevant indicators for early depression, with the Random Forest (RF) model demonstrating the best performance, achieving the highest levels in F1 score (0.94), recall (0.95), negative predictive value (0.92), sensitivity (0.95), and AUC (0.975), indicating its reliability for this classification task. To further interpret the model, we used SHAP plots to identify the key variables influencing depression risk. The results showed that S100β and NSE were the most important variables, followed by PLT.

This study also found significant differences in depression incidence across different countries and regions with varying SDI, particularly in high-SDI areas. In economically developed regions, factors such as intense competition, high levels of academic and work-related stress, high living costs, and fast-paced lifestyles may contribute to the onset of depression, especially in the United States, where these issues have caused a substantial societal burden (22).The possible contributing factors to depression are as follows:1. Academic and Employment Pressure: In high-SDI regions, especially in cities with intense educational competition, students often face high-intensity study tasks and anxiety about future employment. This pressure leads to a high incidence of depression among adolescents.2. Impact of Social Media: The widespread use of smartphones, especially among adolescents, and the prolonged immersion in the virtual world result in a lack of real-life social interactions, which leads to feelings of loneliness, anxiety, and emotional distress, potentially triggering depression.3. Work pressure and career competition: In high-SDI regions, the pressure of career advancement, long working hours, and the fear of unemployment make work stress a significant factor in the development of depression (23). 4.Excessive information consumption: In the era of short videos, adolescents often lack the ability to distinguish false information, which causes a significant psychological gap and leads to feelings of inferiority and a self-destructive mentality.

Meanwhile, low-SDI regions face a lack of medical resources and insufficient awareness and education about depression, leading to underreporting and data imbalances. Some studies suggest that individuals with lower educational attainment are more prone to depression (24, 25), while higher education levels are associated with improved socioeconomic status, better employment and income opportunities, and greater attention to physical health, all of which contribute to a reduced incidence of depression (25, 26). However, these studies often overlook the educational stages, particularly during high school and university, when adolescents are at a heightened risk for depression (27, 28).

With the continued increase in the burden of depression among the youth population and the influence of cultural and economic differences across countries, a single measurement tool may not accurately assess depressive symptoms. In this study, we employed machine learning methods to identify the most important risk factors for depression, with S100β and NSE emerging as key indicators. Several studies have shown that S100β and NSE are biomarkers of neuronal damage (29, 30). Additionally, S100β is associated with brain neuroinflammation, such as in conditions like epilepsy (31, 32). A substantial body of evidence suggests that depression is not only a mental health disorder but also involves neuroinflammation, as well as damage to neurons and glial cell functions (33). Both genetic and environmental factors are important contributors to depression. The destruction and activation of microglia trigger neuroinflammation, which may be a crucial pathological mechanism of depression (34, 35).Microglial cells regulate neuronal function and survival through direct interactions with neurons (36) and the release of cytokines such as IL-1β and IL-6 (37). Additionally, activated microglia alter the brain’s microenvironment by releasing molecules that influence neuronal activity (8). These changes ultimately result in neuronal damage. Upon cellular injury, S100β protein is released (38). S100β interacts with pattern recognition receptors (PRRs) on immune cells, activating the nuclear factor kappa-light-chain-enhancer of activated B cells (NF-κB) signaling pathway (39), which further promotes microglial activation, driving them into a pro-inflammatory state. This leads to the release of additional inflammatory cytokines, exacerbating the immune response and intensifying neuronal damage.

We also identified the importance of PLT in depression and found that PLT levels were significantly higher in patients with depression compared to those without. Some studies suggest that patients with depression have lower levels of Omega-3 polyunsaturated fatty acids, and Omega-3 fatty acids may aid in the treatment of depression (40, 41). Omega-3 polyunsaturated fatty acids reduce and improve neuroinflammation by modulating arachidonic acid (AA) and cytokines. The main metabolite of arachidonic acid is thromboxane A2, which promotes platelet aggregation and vasoconstriction.

Overall, this study reveals the increasing trend in the incidence and DALYs of depression among individuals aged 10–24 globally, and through machine learning methods, we identified key risk factors for depression, such as S100β, NSE, and PLT, providing simple and effective auxiliary tools for the early detection of depression. Therefore, we recommend that biomarkers such as S100β and NSE be included in routine screening for adolescent depression, alongside traditional psychological screening tools. This approach could enhance diagnostic accuracy and facilitate earlier interventions, ultimately improving the mental health of the adolescent population.

However, this study has several limitations. First, global data were sourced from the GBD database, but differences in geography, culture, and economic levels across regions may lead to imbalances in the attention given to and reporting of depression, potentially affecting the representativeness of the data. Second, the clinical data were obtained from a single hospital, which raises concerns regarding the generalizability of the findings; therefore, further multi-center studies are needed to validate the results. Additionally, the limited sample size may increase the risk of overfitting in the machine learning model, which could affect its generalization ability. Future research should address potential overfitting issues and conduct external validation to enhance the robustness and applicability of the model.

In conclusion, our study indicates that depression rates among the global youth population aged 10–24 are on the rise. Additionally, elevated levels of S100β, NSE, and PLT are identified as risk factors for the development of depression.

## Data Availability

The raw data supporting the conclusions of this article will be made available by the authors, without undue reservation.
