# Supplementary material for: The burden of depressive disorder among the global 10–24 age group and the construction of an early risk factors model
Source: Front Psychiatry. 2025 Jun 16;16:1594074. doi: 10.3389/fpsyt.2025.1594074 (PMC12206775; doi:10.3389/fpsyt.2025.1594074)
Supplement: Supplementary file 1 [file Table1.docx]

| **Table S1 The Incidence and DALYs Rates for depressive disorders(10-24 year) in 204 countries** | | |
| --- | --- | --- |
| **Categories** | Incidence | DALY |
| American Samoa | 2480.45(1621.21-3605.12) | 363.04(218.52-549.63) |
| Antigua and Barbuda | 3471.18(2257.2-5325.75) | 505.33(287.22-811.69) |
| Arab Republic of Egypt | 5367.46(3488.38-7972.94) | 775.68(457.33-1243.86) |
| Argentine Republic | 5235.78(3711-7128.06) | 747.99(451.92-1105.78) |
| Australia | 7643.97(5156.89-10690.09) | 1085.17(681.58-1631.45) |
| Barbados | 3969.4(2549.62-5947.81) | 569.89(338.27-916.04) |
| Belize | 3850.67(2542.97-5585.7) | 549.96(325.21-844.76) |
| Bermuda | 3937.22(2490.81-5839.03) | 566.4(320.12-898.07) |
| Bolivarian Republic of Venezuela | 3050.91(2018.86-4451.64) | 435.75(256.62-665.29) |
| Bosnia and Herzegovina | 3360.37(2165.83-4922.23) | 500.96(286.97-777.6) |
| Brunei Darussalam | 2533.11(1664.69-3760.92) | 386.76(226.37-609.44) |
| Burkina Faso | 3269.83(2105.48-4817.74) | 487.04(274.92-757.33) |
| Canada | 6562.29(4335.79-9263.96) | 941.79(557.24-1475.44) |
| Central African Republic | 6356.57(4103.12-9594.39) | 884.65(513.84-1389.66) |
| Commonwealth of Dominica | 3594.52(2338.82-5340.1) | 516.35(286.18-807.49) |
| Commonwealth of the Bahamas | 3574.07(2316.76-5373.96) | 513.83(293.85-834.79) |
| Cook Islands | 3349.29(2033.92-5195.68) | 487.22(266.48-815.07) |
| Czech Republic | 3174.64(2082.58-4610.82) | 467.66(264.6-718.73) |
| Democratic People's Republic of Korea | 1720.49(1163.66-2508.54) | 269.81(160.92-420.1) |
| Democratic Republic of Sao Tome and Principe | 3135.61(1989.38-4617.78) | 473.68(275.39-748.28) |
| Democratic Republic of the Congo | 5817.85(3799.97-8616.11) | 810.86(467.63-1253.29) |
| Democratic Republic of Timor-Leste | 3237.73(2057.55-4710.76) | 477.57(275.24-748.41) |
| Democratic Socialist Republic of Sri Lanka | 4147.08(2796.74-5912.96) | 598.76(373.35-955.87) |
| Dominican Republic | 4081.12(2644.32-6111.99) | 583.93(330.58-924.71) |
| Eastern Republic of Uruguay | 6058.83(3964.31-8689.33) | 858.49(511.95-1387.4) |
| Federal Democratic Republic of Ethiopia | 3935.26(2858.68-5431.84) | 580.18(371.71-856.28) |
| Federal Democratic Republic of Nepal | 4828.97(3077.69-7281.19) | 690.07(403.28-1086.81) |
| Federal Republic of Germany | 5722.2(3670.64-8254.62) | 835.13(509.59-1323.71) |
| Federal Republic of Nigeria | 2595.36(1928.44-3512.09) | 392.3(253.87-572.1) |
| Federal Republic of Somalia | 4768.91(2975.76-6954.32) | 690.85(392.96-1052.28) |
| Federated States of Micronesia | 3003.66(2028.8-4437.2) | 440.19(263.44-711.33) |
| Federative Republic of Brazil | 4550.9(3411.43-6120.56) | 634.83(407.79-940.86) |
| French Republic | 5912.06(3880.62-8502.17) | 857.63(491.53-1331.08) |
| Gabonese Republic | 6801.38(4496.4-10043.59) | 949.82(550.28-1509.35) |
| Georgia | 3911.44(2492.59-5652.59) | 570.92(324.97-898.15) |
| Grand Duchy of Luxembourg | 4983.41(3303.23-7147.59) | 737.26(422.06-1131.62) |
| Greenland | 15273.51(10282.59-21274.47) | 2114.78(1289.81-3263.91) |
| Grenada | 3960.85(2565.73-5933.55) | 570(331.64-906.11) |
| Guam | 4388.71(2895.75-6361.48) | 630.82(362.22-995.23) |
| Hashemite Kingdom of Jordan | 5383.69(3502.71-7881.02) | 780.69(463.72-1232.74) |
| Hellenic Republic | 8688.18(5748.45-13043.77) | 1239.18(715.23-1961.31) |
| Hungary | 2854.68(1854.43-4087.73) | 431.1(245.7-667.83) |
| Independent State of Papua New Guinea | 3133.71(2023.84-4567.24) | 453.87(258.74-719.3) |
| Independent State of Samoa | 2864.95(1850.58-4259.05) | 417.63(244.87-669.44) |
| Ireland | 7861.36(5130.08-11492.96) | 1106.62(682.25-1708.71) |
| Islamic Republic of Afghanistan | 6870.35(4477.77-10123.27) | 962.59(549.32-1532.47) |
| Islamic Republic of Iran | 7033.84(4992.79-9615.22) | 985.14(621.22-1478.42) |
| Islamic Republic of Mauritania | 2856.18(1820.29-4347.97) | 433.14(242.14-686.85) |
| Islamic Republic of Pakistan | 2973.25(2088.96-4123.99) | 434.74(274.01-649.64) |
| Jamaica | 3733.82(2329.03-5696.1) | 540.35(312.53-879.46) |
| Japan | 3906.72(2947.24-5140.75) | 566.81(359.02-833.6) |
| Kingdom of Bahrain | 7113.09(4503.56-10288.95) | 1025.16(577.58-1617.44) |
| Kingdom of Belgium | 5449.72(3605.55-7814.29) | 790.65(459.47-1239.69) |
| Kingdom of Bhutan | 2700.19(1746.28-3960.62) | 413.24(233.15-658.37) |
| Kingdom of Cambodia | 3679.19(2421.35-5356.15) | 537.33(311.83-859.25) |
| Kingdom of Denmark | 5023.6(3297.72-7345.3) | 741.28(433.05-1144.36) |
| Kingdom of Eswatini | 6129.56(3879.67-8951.12) | 869.44(494.29-1375.34) |
| Kingdom of Lesotho | 7775.92(5027.47-11181.03) | 1088.01(622.4-1647.96) |
| Kingdom of Morocco | 7120.74(4608.85-10342.45) | 1014.37(581.29-1640.75) |
| Kingdom of Norway | 5739.65(4177.68-7892.83) | 829.68(525.92-1220.22) |
| Kingdom of Saudi Arabia | 5567.21(3809.56-8141.65) | 820.41(482.81-1283.71) |
| Kingdom of Spain | 7530.13(5253.15-10679.91) | 1090.91(665.07-1724.88) |
| Kingdom of Sweden | 6591.4(4590.47-9011.29) | 938.14(575.52-1443.55) |
| Kingdom of Thailand | 2905.72(1838.57-4125.96) | 437.68(247.29-693.84) |
| Kingdom of the Netherlands | 5872.82(3864.64-8481.89) | 858.64(507.72-1377.28) |
| Kingdom of Tonga | 2704.13(1791.1-4021.42) | 397.28(237.12-616.76) |
| Kyrgyz Republic | 3923.19(2586.85-5609.23) | 569.63(311.95-907.43) |
| Lao People's Democratic Republic | 3336.81(2204.92-4972.56) | 491.58(288.94-778.45) |
| Lebanese Republic | 8196.68(5299.39-12092.52) | 1150.33(641.4-1807.03) |
| Malaysia | 3965.2(2549.16-5803.66) | 583.81(327.24-911.89) |
| Mongolia | 3864.06(2620.33-5549.3) | 560.27(325.26-900.32) |
| Montenegro | 3473.97(2224.08-5033.72) | 512.59(292.52-780.93) |
| New Zealand | 6672(4754.5-9413.53) | 950.38(591.67-1408.84) |
| North Macedonia | 3416.06(2290.78-4967.84) | 509.29(294.03-794.86) |
| Northern Mariana Islands | 3455.47(2304.14-4957.78) | 500.01(292.71-776.9) |
| Palestine | 9287.97(6090.49-13597.09) | 1311.01(765.05-2081.03) |
| People's Democratic Republic of Algeria | 5266.86(3335.84-7945.27) | 765.02(430.53-1233.59) |
| People's Republic of Bangladesh | 4427.93(2928.32-6318.75) | 636.84(370.69-979.71) |
| People's Republic of China | 1387.39(1042.41-1802.23) | 218.45(141.05-316.71) |
| Plurinational State of Bolivia | 4276.53(2773.15-6386.66) | 604.28(345.26-973.04) |
| Portuguese Republic | 7762.13(5010.98-11200.98) | 1115.57(620.25-1745.9) |
| Principality of Andorra | 6046.89(3801.67-9031.76) | 879.56(509.17-1361.34) |
| Principality of Monaco | 6868.64(4253.83-10227.86) | 990.1(540.99-1579.53) |
| Puerto Rico | 2987.45(1976.37-4421.96) | 439.79(253.04-686.48) |
| Republic of Albania | 3100.49(2045.5-4760.2) | 468.98(276.5-730.23) |
| Republic of Angola | 6401.27(4111.77-9490.08) | 889.14(491.88-1378.46) |
| Republic of Armenia | 3676.3(2380.31-5235.8) | 538.19(322.96-844.96) |
| Republic of Austria | 4560.71(2994.07-6645.29) | 680.1(404.29-1082.76) |
| Republic of Azerbaijan | 3112.61(2029.52-4481.34) | 463.2(274.16-736.42) |
| Republic of Belarus | 4943.03(3313.99-7070.46) | 699.76(404.58-1022.24) |
| Republic of Benin | 3511.56(2308.13-5168.83) | 518.29(306.39-818.1) |
| Republic of Botswana | 4844.46(3166.33-7133.73) | 701.74(400.04-1079.36) |
| Republic of Bulgaria | 3459.71(2249.24-4925.45) | 506.52(292.19-774.74) |
| Republic of Burundi | 4268.36(2694.41-6306.8) | 628.64(349.75-975.68) |
| Republic of Cabo Verde | 5006.51(3284.44-7397.13) | 730.35(413.07-1146.15) |
| Republic of Cameroon | 3615.64(2330.94-5415.12) | 531.92(302.64-827.38) |
| Republic of Chad | 3940.79(2472.92-5851.9) | 571.57(337.83-904.05) |
| Republic of Chile | 8063.77(5291.54-11434.39) | 1136.21(656.18-1719.69) |
| Republic of Colombia | 2440.38(1615.75-3530.69) | 363.6(213.66-558.86) |
| Republic of Costa Rica | 3827.8(2523.73-5773.06) | 548.55(313.23-877.67) |
| Republic of Croatia | 3242.69(2130.98-4703.27) | 486.28(287.64-761.79) |
| Republic of Cuba | 4253.91(2838.62-6277.21) | 609.26(369.57-925.46) |
| Republic of Cyprus | 4893.94(3156.32-7310.55) | 729.74(427.24-1140.62) |
| Republic of C么te d'Ivoire | 2826.61(1827.84-4183.04) | 427.17(253.69-668.55) |
| Republic of Djibouti | 4013.36(2557.06-5871.52) | 602.13(355.08-951.25) |
| Republic of Ecuador | 4580.07(2978.15-6628.45) | 653.56(376.81-1046.27) |
| Republic of El Salvador | 4274.39(2817.25-6275.22) | 607.98(351.47-966.03) |
| Republic of Equatorial Guinea | 6348.63(4150.54-9564.61) | 893.2(532.68-1449.44) |
| Republic of Estonia | 4218.58(2671.2-6174.8) | 607.32(369.95-932.47) |
| Republic of Fiji | 3353.08(2254.74-4838.78) | 485.96(293.23-790.35) |
| Republic of Finland | 7613.8(4839.52-10877.51) | 1095.7(670.93-1687.78) |
| Republic of Ghana | 3703.04(2394.17-5665.17) | 549.33(319.42-873.55) |
| Republic of Guatemala | 4093.35(2622.94-6196.86) | 578.45(335.13-921.37) |
| Republic of Guinea | 3560.62(2291.68-5230.71) | 522.92(297.26-809.05) |
| Republic of Guinea-Bissau | 3562.11(2292.39-5219.65) | 524.37(305.17-843.36) |
| Republic of Guyana | 7583.57(5017.17-11273.62) | 1047.47(606.57-1675.78) |
| Republic of Haiti | 4042.88(2616.91-6065.36) | 564.93(324.38-891.41) |
| Republic of Honduras | 3441.09(2209.45-5030.64) | 491.6(269.6-783.99) |
| Republic of Iceland | 4081.92(2651.91-5838.93) | 612.06(357.66-944.23) |
| Republic of India | 3426.87(2534.76-4615.06) | 501.57(315.65-733.28) |
| Republic of Indonesia | 2685.96(1937.23-3637.61) | 402.25(254.17-587.68) |
| Republic of Iraq | 4843.51(3180.94-7145.34) | 700.64(424.77-1095.95) |
| Republic of Italy | 6010.15(4360.02-8015.36) | 865.31(549.17-1283.95) |
| Republic of Kazakhstan | 3850.7(2571.99-5552.68) | 555.9(337.02-868.71) |
| Republic of Kenya | 3809.03(2831.94-5047.77) | 563.76(364.8-819.39) |
| Republic of Kiribati | 3041.45(2027.86-4492.29) | 440.86(259.17-693.12) |
| Republic of Korea | 3388.63(2305.81-4734.03) | 508.27(313.56-782.97) |
| Republic of Latvia | 4288.92(2771.86-6296.36) | 612.71(357.16-949.61) |
| Republic of Liberia | 3623.34(2280.18-5306.75) | 524.88(302.9-822.6) |
| Republic of Lithuania | 5460.51(3626.43-7914.99) | 781.75(466.37-1252.94) |
| Republic of Madagascar | 4322.24(2796.24-6520.45) | 636.19(356.65-1027.23) |
| Republic of Malawi | 3775.21(2418.22-5529.68) | 562.78(335.46-859.74) |
| Republic of Maldives | 3599.61(2278.15-5104.33) | 528.76(313.52-813.96) |
| Republic of Mali | 2682.94(1763.56-3917.16) | 405.47(237.63-634.92) |
| Republic of Malta | 4803.28(3094.58-7113.94) | 715.94(420.53-1124.65) |
| Republic of Mauritius | 6214.95(4066.81-9008.15) | 896.55(510.76-1456.76) |
| Republic of Moldova | 3444.54(2256.48-5019.92) | 507.82(302.6-807.89) |
| Republic of Mozambique | 4623.29(2956.79-6828.45) | 665.51(377.53-1061.32) |
| Republic of Namibia | 4550.04(2941.39-6532.09) | 661.51(380.88-1023.38) |
| Republic of Nauru | 3261.51(1994.72-4946.31) | 471.63(263.84-783.35) |
| Republic of Nicaragua | 4049.85(2673.37-5775.05) | 577.29(346.8-892.13) |
| Republic of Niue | 3260.16(2024.64-5081.95) | 471.29(263.64-747.56) |
| Republic of Palau | 3315.56(2076.47-5084.11) | 480.93(267.91-787.64) |
| Republic of Panama | 3389.97(2186.3-5117.32) | 487.51(277.26-756.94) |
| Republic of Paraguay | 4850.36(3251.43-7063.07) | 680.8(400.62-1078.55) |
| Republic of Peru | 2657.13(1783.16-3949.14) | 392.1(236.51-610.58) |
| Republic of Poland | 2276.63(1653.04-3138.84) | 347.11(224.75-502.47) |
| Republic of Rwanda | 4771.17(3094.36-7083.07) | 704.77(412.31-1140.21) |
| Republic of San Marino | 7022.71(4400.28-10826.65) | 1011.27(554.5-1607.17) |
| Republic of Senegal | 3474.94(2293.06-5146.23) | 512.9(296.08-812.23) |
| Republic of Serbia | 2838.14(1860.8-4198.4) | 427.09(251.78-670.9) |
| Republic of Seychelles | 3437.4(2189.8-5049.75) | 505.65(279.65-794.64) |
| Republic of Sierra Leone | 3266.63(2103.84-4806.73) | 490.62(279.45-793.3) |
| Republic of Singapore | 3575.78(2464.76-4911.94) | 524.57(316.76-797.19) |
| Republic of Slovenia | 3261.8(2145.48-4744.85) | 481.2(281.33-741.64) |
| Republic of South Africa | 5087.43(3794.87-6659.12) | 722.81(451.38-1052.02) |
| Republic of South Sudan | 3790.91(2431.92-5491.79) | 557.45(322.18-871.43) |
| Republic of Sudan | 6009.19(3820.48-8846.96) | 857.74(487.64-1313.56) |
| Republic of Suriname | 7453.15(5021.77-10971.11) | 1027.83(619.55-1674.52) |
| Republic of Tajikistan | 3070.11(2033.69-4415.15) | 456.13(269.05-723.64) |
| Republic of the Congo | 6326.86(3938.63-9520.42) | 884.73(508.48-1395.02) |
| Republic of the Gambia | 4963.57(3182.28-7324.32) | 714.77(404.23-1119.81) |
| Republic of the Marshall Islands | 3012.45(1978.9-4435.76) | 438.62(260.04-681.57) |
| Republic of the Niger | 2933.38(1868.07-4323.29) | 439.2(251.6-683.76) |
| Republic of the Philippines | 3404.24(2487.12-4635.81) | 494.75(316.83-729.69) |
| Republic of the Union of Myanmar | 2444.77(1549.16-3702.06) | 369.41(217.48-595.77) |
| Republic of Trinidad and Tobago | 5776.82(3831.82-8490.99) | 804.52(472.48-1290.58) |
| Republic of Tunisia | 8982.63(5937.81-13157.84) | 1277.65(736.04-2046.56) |
| Republic of Turkey | 6625.49(4145-9489.03) | 959.44(541.15-1526.22) |
| Republic of Uganda | 6489.82(4020.46-9515.8) | 927.05(526.29-1444.16) |
| Republic of Uzbekistan | 3438.63(2280.6-5198.4) | 507.45(307.46-774.75) |
| Republic of Vanuatu | 3217.79(2039.83-4743.16) | 465.25(266.26-726.25) |
| Republic of Yemen | 5422.13(3458.02-7843.46) | 766.24(435.65-1238.65) |
| Republic of Zambia | 3840.58(2416.64-5617.43) | 570.1(332.27-912.35) |
| Republic of Zimbabwe | 2836.09(1811.88-4181.74) | 429.5(255.27-679.29) |
| Romania | 3115.74(2056.29-4498.74) | 461.88(280.32-714.05) |
| Russian Federation | 3591.11(2590.82-4881.82) | 514.01(329.06-765.1) |
| Saint Kitts and Nevis | 4317.17(2604.96-6872.69) | 616.81(342.32-996.13) |
| Saint Lucia | 4563.32(2999.12-6845.96) | 651.16(372.19-1033.35) |
| Saint Vincent and the Grenadines | 4195.73(2764.46-6200.82) | 597.99(353.5-939.67) |
| Slovak Republic | 3112.95(2050.93-4548.58) | 463.06(274.72-729.69) |
| Socialist Republic of Viet Nam | 2435.86(1581.23-3572.89) | 365.07(207.29-574.68) |
| Solomon Islands | 3080.83(2007.69-4620.37) | 446.05(263.42-685.46) |
| State of Eritrea | 4104.58(2648.8-6108.96) | 612.94(362.27-935.75) |
| State of Israel | 6324.14(4086.12-9206.75) | 918.63(549.31-1439.42) |
| State of Kuwait | 4880.83(3048.8-7329.05) | 720.2(401.58-1151.8) |
| State of Libya | 6006.97(3972.04-8877.35) | 865.79(521.47-1363.95) |
| State of Qatar | 5393.38(3462.32-8130.85) | 797.46(445.76-1312.84) |
| Sultanate of Oman | 5822.96(3603.66-8493.37) | 840.52(453.37-1328.55) |
| Swiss Confederation | 5495(3634.38-7896.45) | 809.21(485.56-1226.28) |
| Syrian Arab Republic | 5870.06(3656.58-8679.96) | 840.47(476.56-1345.57) |
| Taiwan (Province of China) | 1604.47(1050.19-2349.61) | 256.68(146.64-399.5) |
| Togolese Republic | 3405.64(2175.43-4890.49) | 504.02(289.74-779.25) |
| Tokelau | 3168.03(1916.48-4802.55) | 457.63(254.24-745.44) |
| Turkmenistan | 3478.6(2283.3-5195.18) | 512.96(300.35-781.96) |
| Tuvalu | 3283.13(2062.35-5069.74) | 477.23(265.64-774.56) |
| Ukraine | 4202.63(2869.07-5872.97) | 597.05(362.01-921.5) |
| Union of the Comoros | 3824.98(2464.4-5738.15) | 577.6(329.53-917.21) |
| United Arab Emirates | 5342.68(3446.03-7912.41) | 773.87(437.71-1261.25) |
| United Kingdom of Great Britain and Northern Ireland | 6656.76(4892.04-8949.97) | 947.77(611.09-1407.56) |
| United Mexican States | 4384.68(3277.74-5868.74) | 616.21(402.24-911.44) |
| United Republic of Tanzania | 4092.17(2657.2-5830.98) | 604.85(350-952.97) |
| United States of America | 10214.88(8022.64-12897.19) | 00 |
| United States Virgin Islands | 3788.28(2500.07-5515.19) | 540.33(311.33-820.58) |
