# Supplementary material for: The burden of depressive disorder among the global 10–24 age group and the construction of an early risk factors model
Source: Front Psychiatry. 2025 Jun 16;16:1594074. doi: 10.3389/fpsyt.2025.1594074 (PMC12206775; doi:10.3389/fpsyt.2025.1594074)
Supplement: Supplementary file 2 [file Table2.docx]

| TableS2 The characteristics of research participants by depressive disorders level | | | | | | | |
| --- | --- | --- | --- | --- | --- | --- | --- |
|  | ALL  N=450 | 0  N=200(44.44%) | level1  N=66(14.67%) | level2  N=79(17.56) | level3  N=17(3.78%) | level4  N=88(15.56%) | *P* |
| Gender: |  |  |  |  |  |  | 0.236 |
| Male | 206 (45.78%) | 100 (50.00%) | 30 (45.45%) | 33 (41.77%) | 10 (58.82%) | 33 (37.50%) |  |
| Female | 244 (54.22%) | 100 (50.00%) | 36 (54.55%) | 46 (58.23%) | 7 (41.18%) | 55 (62.50%) |  |
| Age | 18.00 (15.00-21.00) | 20.00 (17.00-22.00) | 16.00 (15.00-17.00) | 16.00 (15.00-18.00) | 18.00 (16.00-21.00) | 16.00 (14.00-18.00) |  |
| NSE | 11.50 (9.70-17.17) | 10.10 (9.11-11.53) | 11.05 (9.68-12.95) | 12.30 (10.10-16.78) | 17.40 (15.97-18.84) | 19.83 (18.34-22.33) | <0.001 |
| S100β | 5.71 (2.86-8.57) | 2.86 (1.79-3.81) | 6.19 (4.88-6.67) | 6.67 (6.19-7.62) | 10.48 (8.57-10.95) | 11.43 (10.95-12.02) | <0.001 |
| FT3 | 4.47 (4.21-4.79) | 4.51 (4.31-4.82) | 4.46 (4.21-4.72) | 4.49 (4.15-4.81) | 4.34 (4.24-5.05) | 4.34 (4.07-4.65) | 0.016 |
| FT4 | 17.79 (2.20) | 17.62 (2.34) | 18.36 (1.94) | 17.79 (2.09) | 18.23 (1.46) | 17.69 (2.22) | 0.161 |
| TSH | 1.87 (1.31-2.42) | 1.72 (1.25-2.44) | 1.86 (1.58-2.15) | 1.92 (1.36-2.52) | 2.20 (1.34-2.78) | 1.89 (1.48-2.40) | 0.612 |
| BUN | 4.43 (3.83-5.15) | 4.62 (4.02-5.28) | 4.25 (3.90-5.14) | 4.47 (3.79-4.84) | 4.18 (3.52-4.81) | 4.09 (3.45-4.72) | 0.001 |
| CRE | 63.00 (54.00-75.00) | 63.00 (55.00-76.00) | 63.00 (54.25-74.75) | 64.00 (55.50-74.00) | 69.00 (59.00-76.00) | 57.00 (49.75-69.25) | 0.015 |
| LDH | 165.00 (148.00-186.00) | 167.00 (149.00-183.25) | 166.00 (151.50-186.75) | 165.00 (148.50-190.00) | 215.00 (167.00-235.00) | 155.00 (142.00-181.00) | 0.003 |
| CK | 88.00 (65.25-118.00) | 90.50 (72.00-118.25) | 88.00 (67.25-110.50) | 90.00 (63.50-125.00) | 118.00 (74.00-134.00) | 75.50 (57.00-109.75) | 0.159 |
| CK-Mb | 11.00 (9.00-13.00) | 11.00 (9.47-13.30) | 11.00 (8.25-14.00) | 11.00 (8.00-13.00) | 10.00 (7.00-13.00) | 10.50 (9.00-13.00) | 0.182 |
| WBC | 6.30 (5.38-7.60) | 5.97 (5.21-7.06) | 6.79 (5.90-8.66) | 6.56 (5.74-7.71) | 6.70 (4.82-7.64) | 6.47 (5.59-7.66) | <0.001 |
| RBC | 4.88 (4.51-5.35) | 4.88 (4.59-5.30) | 5.02 (4.62-5.51) | 4.85 (4.49-5.20) | 5.00 (4.50-5.35) | 4.74 (4.50-5.36) | 0.456 |
| HGB | 138.00 (129.00-151.00) | 140.00 (133.00-152.00) | 138.50 (131.00-153.50) | 137.00 (125.00-148.50) | 145.00 (131.00-158.00) | 136.00 (123.00-151.00) | 0.022 |
| PLT | 264.00 (226.00-303.00) | 256.50 (216.00-286.00) | 298.50 (252.25-346.75) | 271.00 (231.00-310.00) | 249.00 (222.00-315.00) | 273.50 (237.00-317.00) | <0.001 |
| Values are expressed as mean ± SD, medians (interquartile ranges), or percentages | | | | | | | |
